# Supplementary figures and images for: Dietary Components Associated with the Risk of Gastric Cancer in the Latin American Population: A Systematic Review and Meta-Analysis
Source: Foods. 2025 Mar 19;14(6):1052. doi: 10.3390/foods14061052 (PMC11942000; doi:10.3390/foods14061052)

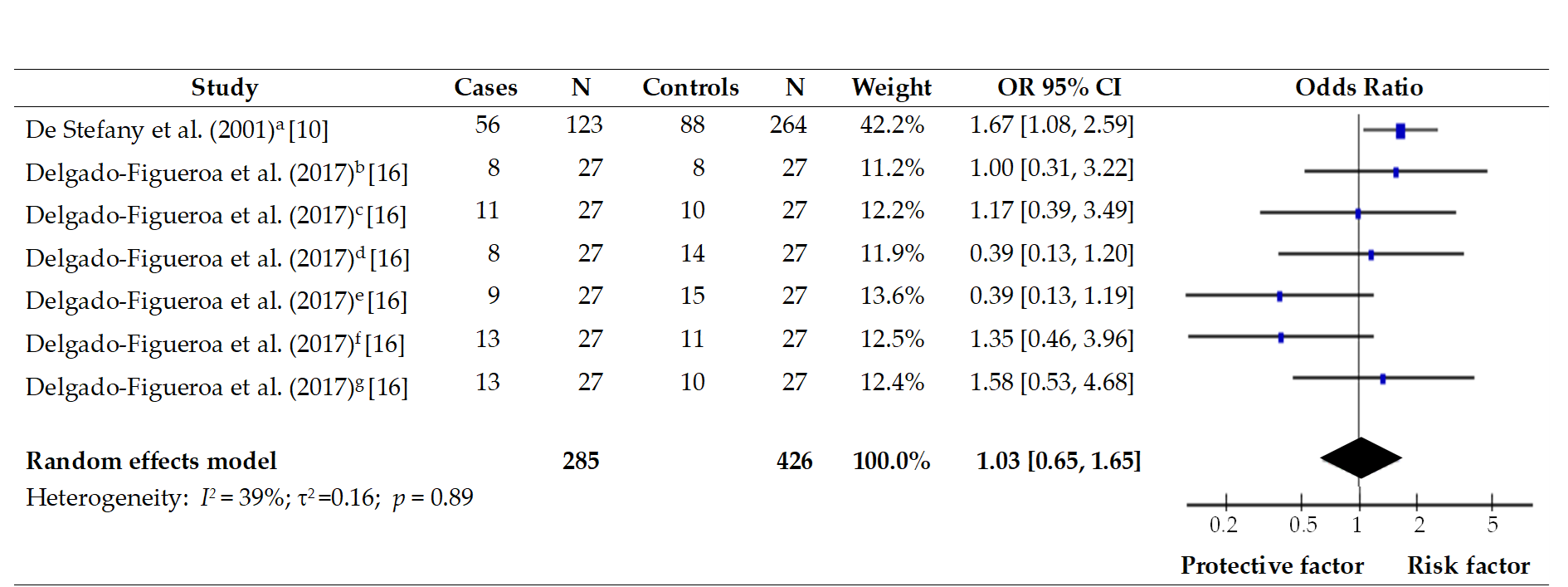

Supplement: Supplementary file 1 [file foods-14-01052-s001.zip › Figure_S1.tif]

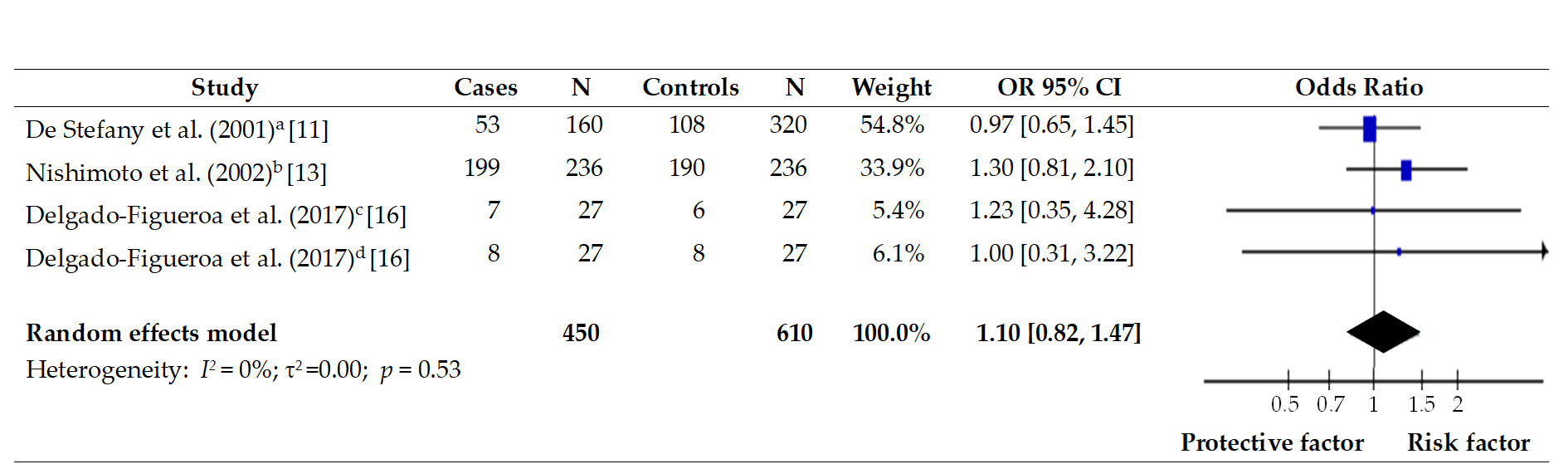

Supplement: Supplementary file 1 [file foods-14-01052-s001.zip › Figure_S2.tif]

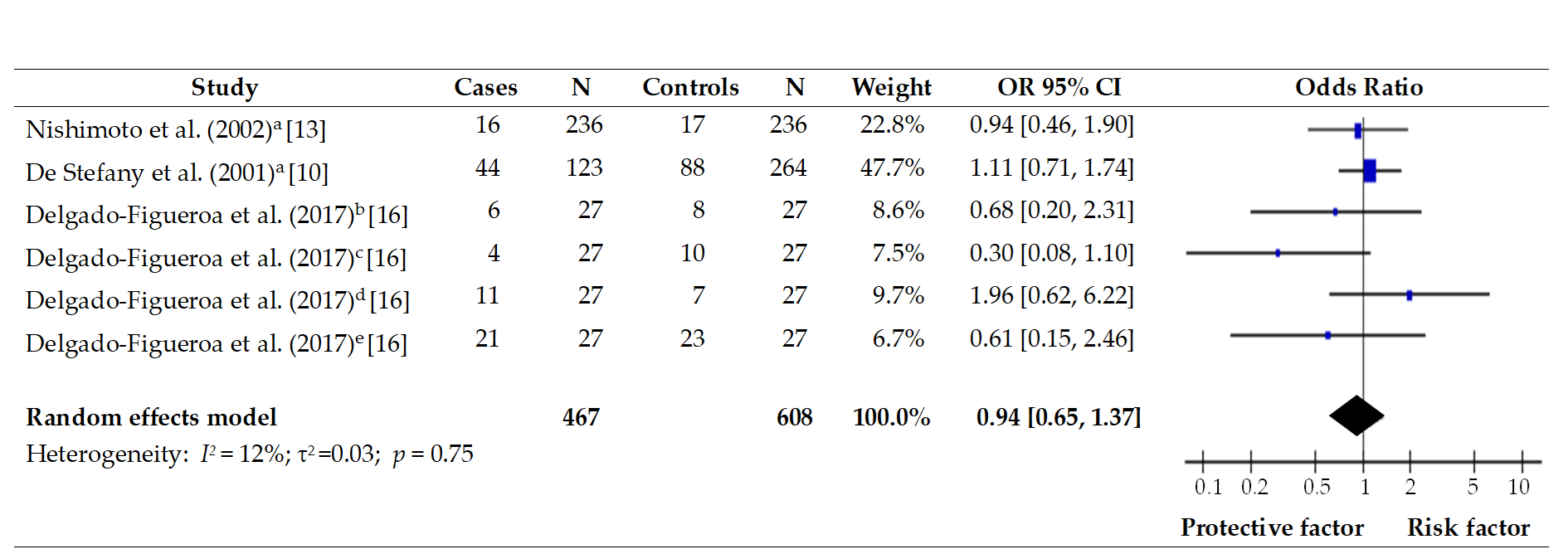

Supplement: Supplementary file 1 [file foods-14-01052-s001.zip › Figure_S3.tif]

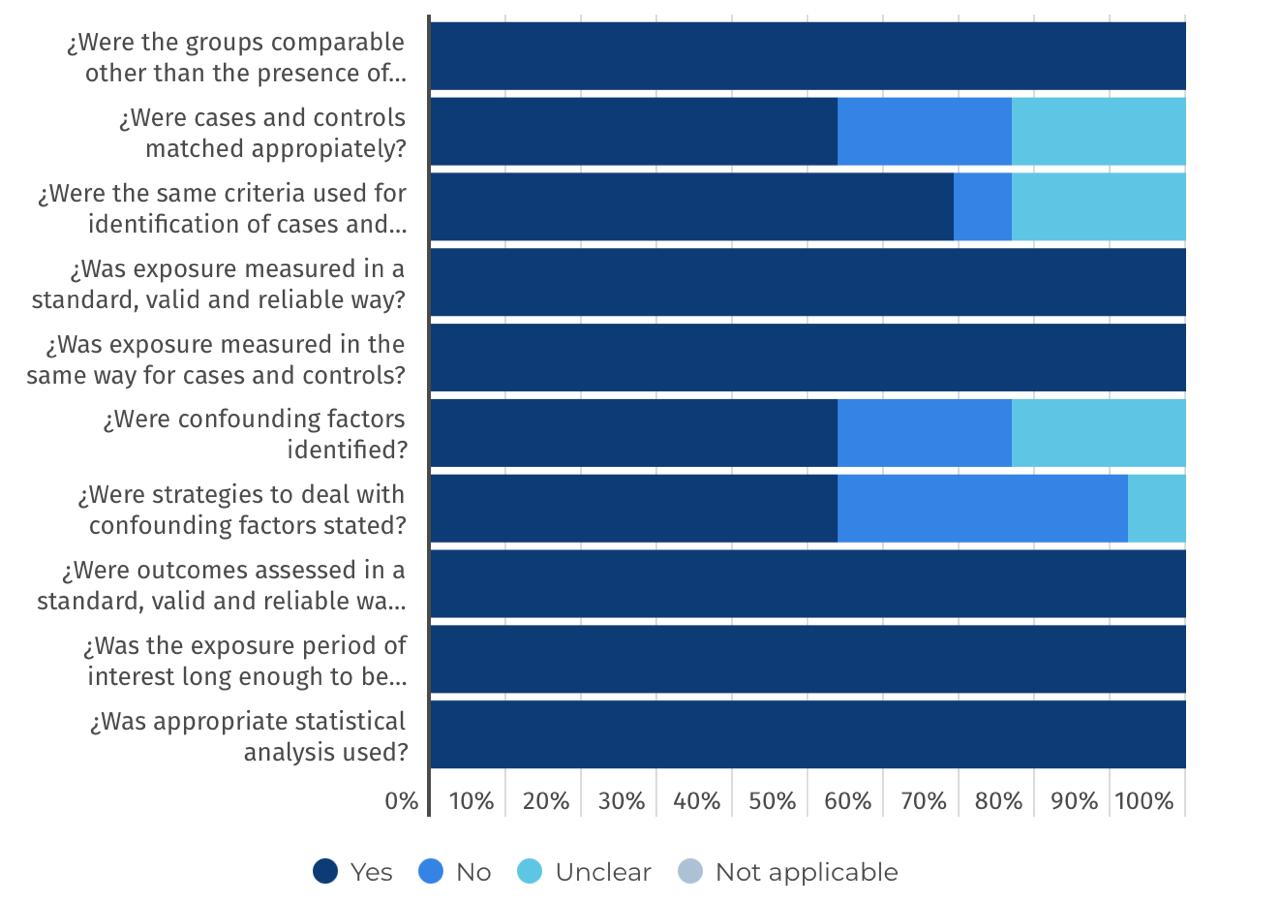

Supplement: Supplementary file 1 [file foods-14-01052-s001.zip › Figure_S4.tif]
